# Radiomics and radiogenomics in gliomas: a contemporary update

**DOI:** 10.1038/s41416-021-01387-w

**Published:** 2021-05-06

**Authors:** Gagandeep Singh, Sunil Manjila, Nicole Sakla, Alan True, Amr H. Wardeh, Niha Beig, Anatoliy Vaysberg, John Matthews, Prateek Prasanna, Vadim Spektor

**Affiliations:** 1 Neuroradiology Division, Department of Radiology, Newark Beth Israel Medical Center, Newark, NJ USA; 2 Department of Neurosurgery, Ayer Neuroscience Institute, The Hospital of Central Connecticut, New Britain, CT USA; 3 Department of Biomedical Engineering, Case Western Reserve University, Cleveland, OH USA; 4 Department of Biomedical Informatics, Stony Brook University, Stony Brook, NY USA; 5 Neuroradiology Division, Department of Radiology, Columbia University Medical Center, New York City, NY USA

**Keywords:** Medical imaging, Cancer

## Abstract

The natural history and treatment landscape of primary brain tumours are complicated by the varied tumour behaviour of primary or secondary gliomas (high-grade transformation of low-grade lesions), as well as the dilemmas with identification of radiation necrosis, tumour progression, and pseudoprogression on MRI. Radiomics and radiogenomics promise to offer precise diagnosis, predict prognosis, and assess tumour response to modern chemotherapy/immunotherapy and radiation therapy. This is achieved by a triumvirate of morphological, textural, and functional signatures, derived from a high-throughput extraction of quantitative voxel-level MR image metrics. However, the lack of standardisation of acquisition parameters and inconsistent methodology between working groups have made validations unreliable, hence multi-centre studies involving heterogenous study populations are warranted. We elucidate novel radiomic and radiogenomic workflow concepts and state-of-the-art descriptors in sub-visual MR image processing, with relevant literature on applications of such machine learning techniques in glioma management.

## Background

Primary brain tumours account for about 2% of all cancers in the US with an incidence of about 23 per 100,000. Gliomas account for 80.6% of all malignant brain tumours.^1^ The incidence is highest for glioblastoma (3.21 per 100,000 population), followed by diffuse astrocytoma (0.46 per 100,000 population). The age-adjusted mortality rate is 4.4 per 100,000 and the 5-year survival rate is 35%.^1^ However, the rate varies significantly by age at diagnosis and the histology of the tumour.

Advances in our understanding of the molecular pathogenesis of gliomas has prompted significant changes to the World Health Organization (WHO) classification of central nervous system (CNS) tumours in 2016.^2^ Previously, the classification criteria was based solely on microscopic features.^3^ The new criteria reclassifies entities with the incorporation of genetic information in certain tumours. These changes were incorporated because of the impact genetic factors have on tumorigenesis and subsequent therapy.

In today’s era of modern imaging, accurate non-invasive prediction of glioma grade/type, survival, and treatment response remains challenging. Stereotactic brain biopsy, despite being invasive and costly, remains the reference standard for histological and genetic classification; however, pathological diagnosis may still remain inconclusive in 7–15% of patients.^4^ This necessitates imaging surrogates to characterise tumour heterogeneity. Recently, multiple studies have shown strong association between morphological features from multiparametric magnetic resonance imaging (MRI) and survival.^5–10^ Similarly, functional imaging techniques such as perfusion weighted MRI and magnetic resonance spectroscopy (MRS) have been shown to be beneficial when used along with morphological features, but with limited success and reproducibility.^11–16^ The limitations in current imaging techniques provide an opportunity for more sophisticated sub-visual feature analysis to augment the morphological features and current functional imaging capabilities.

Radiomics refers to the computerised extraction of quantifiable data from radiological images in the form of radiographical cues that are usually sub-visual.^17,18^ The extraction of these data creates mineable databases from radiological images which can be used for diagnosis, prognosis characterisation, and to assess or predict response to certain therapies.^19–21^ Genetic mutations often determine the aggressiveness of the tumour and have been shown to be associated with a lesion’s growth pattern and response to therapy. Radiomic features have been shown to identify genomic alterations within tumour DNA and RNA. The integrated study of data from radiographical and the genomic scales is termed radiogenomics.

In this review, we describe the applications of radiomics and radiogenomics from the perspective of neuroradiologists, neurosurgeons, and neuro-oncologists. Specifically, we review work that highlights the importance of the evolving field for diagnosing and predicting prognosis of individuals with different brain tumour types. Additionally, we discuss the potential and importance of integrating these applications into radiological workflows to improve patient care and outcome.

## Overview of radiomics and radiogenomics workflow

**Fig. 1:**
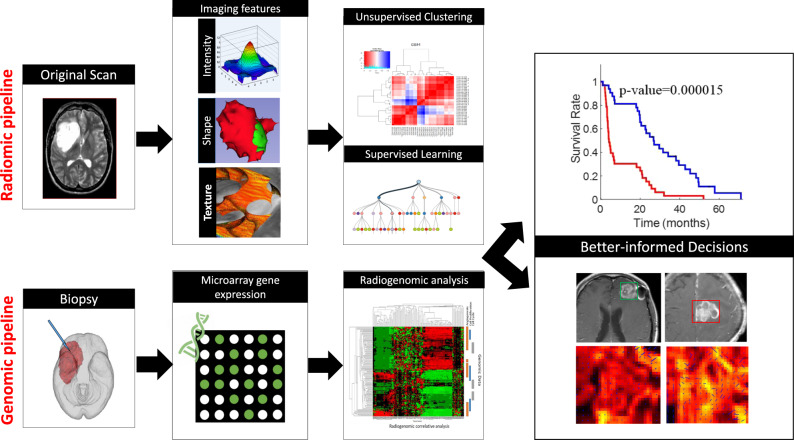
Radiomics and Radiogenomics workflow. Use of Radiomics and Radiogenomics pipelines in personalized medicine.

Radiomics is an emergent field that involves converting radiological images into high-dimensional mineable data in a high-throughput fashion. This multi-step process involves (a) image acquisition and reconstruction, (b) image pre-processing, (c) identification of regions of interest, (d) feature extraction and quantification, (e) feature selection, and (f) building predictive and prognostic models using machine learning (Fig. 1).^22^ To account for MRI intensity non-uniformity, inter- and intra-site scanner variability, image processing routines, such as intensity normalisation, voxel intensity calibration and bias field correction, are used as a precursor to radiomic feature extraction.^23–25^ The segmentation of the regions of interest (ROI) can be achieved by manual, semi-automated or fully-automated methods.^19,26–29^ Radiomic features are then extracted from the identified ROIs. Common features can be divided into the following groups: morphological radiomics, textural radiomics, and functional radiomics.

Following feature extraction, different statistical methods are used to select a subset of top features that correlate with the expected outcome.^30^ Commonly used feature selection algorithms include minimum redundancy maximum relevance (mRMR) algorithm^31,32^ and sequential feature selection methods.^33^ Feature selection is performed in order to reduce potential model overfitting associated with the high dimensionality of the radiomic feature set. Once top features are identified, machine learning classifiers and other statistical methods such as the Cox-proportional Hazards modelling techniques^34^ are used to build predictive and prognostic models. Sanduleanu et al. proposed a “radiomics quality score” tool to assess the quality of the radiomics research study linking tumour biology; however, interpretability of the outcomes of those scores is still questionable.^35^

The recent advent of radiogenomics has also accelerated the integration of multi-omic data for accurate diagnosis and improved personalised cancer treatments. The first step of the radiogenomic pipeline in neuro-oncology (Fig. 1) is to acquire genomic material via a fresh frozen paraffin embedded (FFPE) sample or a tissue microarray (TMA) sample obtained from a stereotactic brain biopsy from within the brain tumour. Next, bioinformatics techniques, such as sequencing, can detect single-gene mutations. For instance, epidermal growth factor receptor (EGFR) amplification, *O*^6^-methylguanine-methyltransferase (MGMT) methylation can be detected by analysing the proteins through immunohistochemical (IHC) analysis and next-generation sequencing (NGS) techniques such as mRNA sequencing. mRNA sequencing, whole-exome sequencing, and whole-genome sequencing can help detect multi-gene expression anomalies. The decisive goal of radiogenomic analysis involves associating gene mutations and pathways directly with distinct imaging phenotypes.

## Radiomic feature groups

### Morphological radiomics

Morphological radiomic features are used to quantify lesion topology induced by the proliferating boundaries. These can be further divided into global and local morphological features. Global features characterise the contour of the lesion by extracting measurements such as roundness, perimeter, diameters of major and minor axes, and elongation factor. Local morphological features characterise the surface curvature attributes derived from isosurfaces.^26,36^ These comprise quantitative measurements such as degree of curvature (curvedness) and degree of sharpness.

### Textural radiomics

#### Structural texture analysis

Structural methods describe texture by identifying structural primitives and their placement rules. Multi-scale, multi-resolution steerable bandpass filters like Gabor filter banks^37,38^ are among the most widely used orientation-based structural descriptors. Gabor descriptors are modelled to mimic the way the human visual system deciphers object appearances, by decomposing the original image into filter responses of a sinusoidal wave of multiple frequencies and orientations. Gabor filters have been shown to distinguish pathologies on histology samples as demonstrated by Doyle et al.^39^

#### Statistical texture analysis

Statistical methods analyse the spatial distribution of grey values by computing local features at each image point and deriving a set of statistics from the distribution of local features. One commonly used statistical technique for identifying shape-based object classes is histogram of oriented gradients (HOG).^40^ Traditionally, the applicability of HOG has been demonstrated for detection of human forms in cluttered images. Multi-coordinate HOG can distinguish different categories of lung tissues in high-resolution tomography images. It characterises local object appearance and shape by computing distribution of local intensity gradients. Grey level co-occurrence matrix (GLCM) features, popularly known as Haralick features^41^ and originally designed for aerial photography, utilise the values of distance and angle for a combination of grey levels.

#### Texture analysis using a combination of statistical and structural techniques

Local binary patterns (LBP) is a textural operator that combines statistical and structural methods in appearance classification. LBP is robust with regards to illumination changes and has been shown to be useful in medical datasets which are corrupted by patient motion artefacts. This feature presents texture information as a joint distribution of the intensity of a central pixel and that of its neighbors.^42^ Li et al.^43^ demonstrated the use of LBP along with neural networks to classify endoscopic images. Another feature that combines statistical and structural techniques is the co-occurrence of local anisotropic gradient orientations (CoLlAGe) descriptor which seeks to capture and exploit local anisotropic differences in voxel-level gradient orientations to distinguish similar appearing phenotypes.^44^

### Functional radiomics

A critical obstacle to the clinical adoption of traditional radiomic features is its low biological interpretability. To qualify as a biomarker, an attribute should not only be measurable and reproducible, but also be reflective of the underlying anatomy or physiology. It is imperative to discover radiomic signatures that are biologically relevant. Functional radiomic markers are a new class of markers which specifically target the issue of ‘interpretability’ by modelling features that directly capture underlying physiological properties such as angiogenesis. Properties of vessels feeding the lesions (such as convolutedness, density) play an important role in the drugs’ ultimate response. Recently, tortuosity-based features capturing local and global disorder in vessel network arrangement have been shown to be effective in diagnosis and treatment response assessment.^45^ Deformation descriptors are another class of functional radiomics markers which seek to measure tissue deformation in the brain parenchyma due to mass effect.^46^ These features provide an insight into the microenvironment outside the visible surgical margins.

Vessel architecture imaging (VAI) MRI is a technique that non-invasively measures parameters to describe structural heterogeneity of brain microvasculature.^47–49^ The different gradient echo (GE) and spin echo (SE) images produce an apparent different variation in the MRI readout based on the structural and physiological properties of the vessels. Stadlbauer et al.^47^ examined gliomas (*n* = 60) using vascular architectural mapping (VAM). They introduced three new VAM biomarkers (i) microvessel type indicator (MTI), (ii) vascular-induced bolus peak time shift (VIPS), and (iii) the curvature (Curv) and adapted known parameters, microvessel radius (RU) and density (NU). MTI and VIPS parameters were helpful in detecting neovascularisation, especially in the tumour core of the HGGs, whereas curvature showed peritumoral vasogenic oedema which correlated with neovascularisation in the tumour core of HGGs. These biomarkers gave insight into complexity and heterogeneity of vascular changes in gliomas to differentiate HGGs versus LGGs.^50^ Furthermore, combining multiparametric quantitative blood oxygenation level-dependent approach (qBOLD) with VAM parameters helped distinguish LGGs versus HGGs and identify isocitrate dehydrogenase (IDH) mutation status with higher sensitivity.^50^ Stadlbauer et al.^51^ also performed analysis of vascular hysteresis loop (VHLs) in combination with the VAM biomarkers to assess response of glioblastoma to anti-angiogenic therapy. MTI was found to be useful to predict responding versus non-responding regions, whereas, Curv was better to assess severity of vasogenic oedema. Price et al.^52^ used diffuse tensor imaging (DTI) with MR perfusion and MRS imaging to determine changes in the invasive versus non-invasive margins of glioblastomas to better predict treatment efficacy and overall survival.^53,54^

### Semantic features

Semantic features, such as tumour location, shape, and geometric properties on structural MRI,^19,55^ are qualitative features used by neuroradiologists to describe the tumour environment. Previous studies have found that semantic features are related to the genetic phenotype of brain tumours.^56^ The Visually AcceSAble Rembrandt Images (VASARI) project by TCIA established a feature set to enable consistent description of gliomas using a set of defined visual features and controlled vocabulary.^57^ Studies have shown that these features are highly reproducible and provide meaningful guidance in glioblastomas.^5^ Semantic features are also robust to changes in image acquisition parameters and noise and can be used along with more sophisticated radiomic features in machine learning settings.^22^

## Diagnostic applications

### Differentiating tumours based on texture analysis

Many studies have shown the application of textural analysis for differentiating HGGs from LGGs. Skogen et al.^58^ applied a filtration-histogram technique for characterising tumour heterogeneity. In a cohort of 95 patients (27-grade II, 34-grade III, and 34-grade IV), by using standard deviation (SD) at a fine texture scale, they were able to distinguish LGGs from HGGs with sensitivity and specificity of 93% and 81% (AUC 0.91, *P* < 0.0001). Tian^59^ et al. applied textural analysis on multiparametric MRI of 153 patients and reported an accuracy of 96.8% for classifying LGGs from HGGs and 98.1% for classifying grade III from grade IV using an SVM classifier. Xie et al.^60^ evaluated five GLCM features from (DCE)-MRI of 42 patients with gliomas. They reported that entropy (AUC = 0.885) and IDM (AUC = 0.901) were able to differentiate grade III from grade IV and grade II from grade III gliomas, respectively.

Suh et al.^61^ used MRI radiomics-based machine-learning algorithms in differentiating central nervous system lymphoma (PCNSL; *n* = 54) from non-necrotic atypical glioblastoma (*n* = 23). Over 6000 multi-sequence and multi-regional radiomic features including shape, volume, and texture were first obtained. AUCs achieved statistical significance for each of the three machines used, demonstrating a higher diagnostic performance than three radiologists.

### Characterising tumour molecular subtypes of low-grade gliomas (LGG)

Adult LGGs with IDH1 mutation are associated with a better prognosis and longer survival than are IDH1-wild-type adult LGGs (i.e., negative for IDH1 mutation), which act much more aggressively.^2^ 1p/19q codeletion has been associated with good response to chemotherapy and a very favourable prognosis. Non-invasive radiogenomic molecular phenotyping may allow for personalised therapeutic decisions in LGG.

Multiple groups have evaluated radiomic features to determine molecular phenotype of gliomas.^62^ Zhang et al.^63^ extracted 15 optimal radiomic features (*n* = 152) using SVM-recursive feature elimination (SVM-RFE) that could detect IDH mutation with accuracy of 82.2%. Han et al.^64^ extracted radiomic features from 42 patients with histopathologically confirmed gliomas. They showed joint variables derived from T1-weighted image (T1WI), T2 weighted image (T2WI), and contrast-enhanced T1WI imaging histograms and GLCM features could be used to detect IDH1-mutated gliomas. The AUC of joint variable_T1WI+C_ for predicting IDH1 mutation was 0.984, and the AUC of joint variable_T1WI_ for predicting the IDH1 mutation was 0.927. Jakola et al.^65^ reported that textural homogeneity could discriminate between LGG patients with IDH mutation and IDH wild-type (*P* = 0.005). The AUC for combined parameters (tumour homogeneity and tumour volume) was 0.940 for predicting IDH mutation. However, this method could not differentiate LGG with IDH mutation with or without 1p19q codeletion. Bahrami et al.^66^ measured tissue heterogeneity and edge contrast (EC) on FLAIR images of 61 patients and reported that patients with IDH wild-type tumours showed higher signal heterogeneity (*P* = 0.001) and lower EC (*P* = 0.008) compared with IDH mutant type. Among patients with IDH mutant tumours, 1p/19q codeleted tumours had greater signal heterogeneity (*P* = 0.002) and lower EC (*P* = 0.005), and MGMT-methylated tumours showed lower EC (*P* = 0.03).

Metabolic alteration of D-2-hydroxyglutarate (D-2HG) production is a hallmark for IDH mutation in gliomas.^67^ Recently, a number of groups have demonstrated reliable detection of D-2HG using in vivo ^1^H MRS.^68–70^ Andronesi et al.^68^ reported apparent in vivo detection of D-2HG using 2D correlation spectroscopy (COSY) and J-difference spectroscopy in IDH1-mutated gliomas. Rohle et al.^71^ identified a selective R132H-IDH1 inhibitor via a high-throughput screening, which in a dose-dependent manner can inhibit the production of R-2HG, providing further avenues for targeted therapies.

### Differentiating treatment effects (radiation necrosis, pseudoprogression) and tumour recurrence

A major challenge in the management of glioblastoma is the difficulty of accurately assessing the response to treatment with several entities that can mimic tumour recurrence or progression on structural MRI, namely pseudoprogression and radiation necrosis (RN).^26,72–74^ Visual diagnosis is often ambiguous and remains extremely challenging, clinically.^75^ Functional MRI, such as MR perfusion and MRS improves the diagnostic accuracy; however, this may not be universally available and are often difficult to reproduce.^72,76–79^ Radiomics provides a non-invasive approach to reliably distinguish tumour recurrence from treatment effects and can potentially help prevent unnecessary biopsies.^26,36,80^

By combining 3D shape and surface radiomic features extracted from both T1WI-enhancing lesions and T2WI/FLAIR hyperintense perilesional area, Ismail et al.^36^ were able to differentiate between true progression and pseudoprogression with 90.2% accuracy (*n* = 105). The two most discriminative features were found to be local features capturing total curvature of the enhancing lesion and curvedness of the T2WI/FLAIR hyperintense peritumoral region. The differential expression patterns may be attributed to the alteration of white matter structure via infiltration, resulting in surface shape irregularities.

Similar to pseudoprogression, distinguishing brain tumour recurrence (RT) from RN can be challenging on routine MRI due to the lack of objective methods of assessment. A texture analysis in conjunction with support vector machine approach presented by Larroza et al.^74^ could differentiate brain metastasis from RN (*n* = 115) with an AUC of >0.9. CoLlAGe features were shown to express differentially across different grades of RN and tumours.^44,81^ A novel aspect of this study was the inclusion of pure cerebral RN from nasopharyngeal carcinoma. CoLlAGe entropy values were found to be skewed toward higher values for the predominant tumour cases compared with the pure cerebral RN or predominant cerebral RN.^44,81,82^ Prasanna et al.^81^ further demonstrated that incorporating CoLlAGe features from pure RN in the training set resulted in improved classification performance of the predominant RN/RT, compared with using features from the predominant RN/RT alone. This potentially demonstrates, to some extent, the similarity in structural and morphological properties between pure RN and its mixed presentations.

Hu et al.^80^ presented an automated technique to identify RN at high spatial resolution using multi-parametric MR features. The classification feature vector comprised eight parameters derived from the multiple sequences, including contrast-enhanced T1, T2, FLAIR, PD, ADC, rCBF, rCBV and MTT. The mean AUC obtained on *n* = 31 (RT = 15, RN = 16) cases was 0.94. Interestingly, the performance using ADC features was significantly better than those using conventional MRI measures. Combining textural measures of heterogeneity with tracer uptake kinetics was shown to be more effective in distinguishing brain metastasis recurrence from radiation injury.^83^ The diagnostic accuracy when using tumour-to-brain ratios (TBRs) of ^18^F-FET uptake was 83% which increased to 85% upon combining with textural parameters such as coarseness, short-zone emphasis, or correlation.^83^ This shows the potential complementary diagnostic information that the texture attributes may provide along with other modalities.

## Prognostic applications

### Survival stratification in glioblastomas

**Fig. 2:**
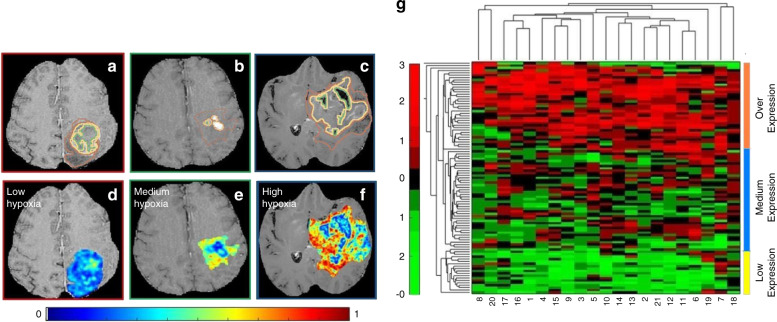
Construction of Hypoxia Enrichment Score. **a**–**c** show a 2D Gd-T1w MRI slice with expert-annotated necrosis (outlined in green), enhancing tumour (yellow) and oedematous regions (brown) in three different GBM patients that exhibited low, medium, and high hypoxia enrichment score (HES) respectively. The corresponding inverse difference moment (Haralick) feature map has been overlaid on the manually annotated tumour regions, for HES_low_ (**d**), HES_medium_ (**e**), and HES_high_ (**f**). **g** Unsupervised clustering of the RNAseq data from the 21 hypoxia associated genes clustered as high hypoxia (HES_high_—shown in navy blue, medium hypoxia (HES_medium_—shown in teal) and low hypoxia (HES_low_—shown in yellow). The *x* axis in the clustergram represents the 21 genes and *y* axis represents the patient population of 97 GBM cases. Figure from Beig et al.^87^; licensed under a Creative Commons Attribution (CC BY) license.

Over 40% of glioblastoma patients do not respond to conventional chemo-radiation therapy and show progression within 6–9 months.^84^ Hypoxia in glioblastoma multiforme is a key pathway known to promote tumour neovascularisation and invasion of healthy tissue as well as driving treatment resistance leading to poor prognosis.^85^ Multiple pathways such as cellular proliferation, apoptosis and increased angiogenesis are also known to contribute towards poor progression-free survival (PFS) outcome. Currently, there is a lack of well-validated biomarkers to monitor levels of hypoxia and predict treatment response to anti-angiogenic agents.^86^ Beig et al.^87^ showed that surrogate radiomic descriptors can capture the extent of hypoxia of glioblastoma on pre-treatment MRI and predict survival (Fig. 2). In this radiogenomic study, the authors used microarray expression data from 85 glioblastoma patients to construct a hypoxia enrichment score (HES). Next, a radiomic model was trained that correlated with HES, and then used to stratify glioblastoma based on their overall survival (OS). On a validation set of *n* = 30 patients, the radiomic features which were strongly associated with HES, could also distinguish short-term survivors (OS < 7 months) from long-term survivors (OS > 16 months) (*P* = 0.003). Another study by Kickingereder et al.^88^ (*n* = 119) extracted over 12,190 radiomic features of glioblastoma and concluded that an 11-feature radiomic signature allowed for the prediction of PFS and OS.

Jain et al.^89^ investigated imaging, genomic, and haemodynamic parameters obtained from the non-enhancing region (NER) of glioblastoma on multi-sequence enhanced MRI. Increased relative cerebral blood volume of the NER (rCBV_NER_) and NER crossing the midline were found to be associated with poor survival. Wild-type EGFR mutation was the only genomic alteration that was associated with significantly poor survival in patients with high rCBV_NER_.

**Fig. 3:**
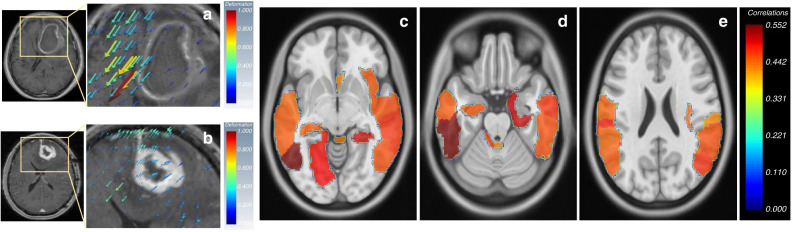
Deformation Radiomics. **a**, **b** Deformation vectors representing tissue displacement are shown as volume rendered 3D quivers overlaid on an image slice of right-hemispheric GBMs. The deformation magnitude is proportional to the size of quivers. Higher value of deformation magnitude is represented by ‘red’ and lower value by “blue” colour respectively. The quivers also show the direction of tissue displacement. **c** The AAL regions in which, the MEDH negatively correlated with survival with *P* < 0.05 for right-hemispheric tumour group (neurological view). The colormaps show the negative correlation values (shown as positive for easier representation). Figure from Prasanna et al.^46^; licensed under a Creative Commons Attribution (CC BY) license.

Prasanna et al.^46^ discussed how mass effect-induced deformation heterogeneity (MEDH) from glioblastoma on multi-sequence MRI affects survival. High expression of MEDH in the areas of language comprehension, social cognition, visual perception, emotion, somatosensory, cognitive and motor-control functions were found to be associated with worse survival (Fig. 3). McGarry et al.^90^ were able to use multi-sequence MRI radiomic profiles (RPs) of newly diagnosed glioblastoma in order to further stratify patient prognosis. Each voxel examined was assigned an RP. Five such RPs were predictive of overall survival prior to therapy initiation.

### Survival stratification in lower-grade gliomas

Liu et al.^91^ developed a radiomics signature to predict PFS in LGGs. The radiomic risk score (RRS) was calculated and the genetic characteristics of the group with high-risk scores were identified by radiogenomic analysis. Biological processes of cell adhesion, cell proliferation, differentiation and angiogenesis were identified to be positively associated with the RRS. A nomogram combining the RRS with other clinical features improved patient stratification and resulted in better assessment of PFS.

Zhou at al.^92^ built radiomic models using automated texture analysis and VASARI features to predict IDH1 mutation (AUC = 0.86), 1p/19q codeletion status (AUC = 0.96), histological grade (AUC = 0.86) and tumour progression (AUC = 0.80) in patients (*n* = 165) with diffuse low- and intermediate-grade gliomas. They found that on MRI images no enhancement and a smooth non-enhancing margin were predictive of longer PFS; smooth non-enhancing margins were also a significant predictor of longer OS in LGGs.

Li et al.^93^ selected nine radiological features that could predict Ki-67 expression level and achieved accuracies of 83.6% and 88.6% in the training (*n* = 78) and validation (*n* = 39) sets, respectively. Only spherical disproportion (SDp) feature was found to be a prognostic factor with patients in the high SDp group. Ki-67 expression level and SDp were independent prognostic factors in the multivariate Cox regression analysis.

## Applications in selecting optimal therapy

### Stratifying anti-angiogenic treatment response for recurrent glioblastomas

Anti-angiogenic treatment is the treatment of choice for recurrent glioblastoma. Kickingereder et al.^94^ investigated imaging biomarkers that may be able to predict treatment outcome. By utilising multi-sequence MRI with recurrent glioblastoma prior to anti-angiogenic treatment with bevacizumab, radiomic MRI features were extracted and analysed from 172 patients. Using these radiomic features, a model was created to predict median PFS and OS in low-risk and high-risk groups. The authors concluded that radiomics might be able to identify patients who would most benefit from bevacizumab therapy.

Bahrami et al.^95^ evaluated 33 HGG patients before and after initiation of bevacizumab treatment. They segmented volume of interest (VOI) within FLAIR hyperintense region and extracted edge contrast (EC) magnitude for each VOI using gradients of the 3D-FLAIR images. They reported that lower EC of the FLAIR hyperintense region was associated with poor PFS (*P* = 0.009) and OS (*P* = 0.022). Other relevant literature has been summarised in Tables 1–3.

**Table 1:** Diagnostic applications.

Author	No. of patients	Magnet strength/MRI Sequences	Segmentation manual vs automatic	Software	Type of radiomic analysis	Best discriminating features	Machine learning/statistical approach	Results
Ismail et al.^36^	105 (Training *n* = 59, test *n* = 46; GBM)	1.5 T/T1-CE, T2WI/FLAIR	Manual 2D	Matlab^a^	30 shape features; 14 “global” contour characteristics and 16 “local” curvature	Top two most discriminative features including the SD of S and the mean of K_T_	SVM	3D shape attributes from the lesion habitat can be differentially expressed across pseudoprogression and tumour progression and could be used to distinguish these radiographically similar pathologies
Larroza et al.^74^	115 (RN = 32, radiation treated mets = 23, untreated mets = 60)	T1-CE	Manual 2D	Mazda	179 features; histogram, gradient, GLCM, GRLM, wavelets	Intensity 90^th^ percentile	SVM	High classification accuracy (AUC > 0.9) was obtained using texture features and a support vector machine classifier to differentiate between brain metastasis and RN
Prasanna et al.^81^	75 (different grades of RN)	T1-CE	Manual 2D	Matlab	Four CoLlAGe entropy	Collage entropy skewness	RF	COLLAGE features exhibited decreased skewness for patients with pure and predominant RN and were statistically significantly different from those in patients with predominant recurrent tumours.
Hu et al.^80^	31 (RT = 15, RN = 16)	T1-CE, T2, FLAIR, PD, ADC, rCBF, rCBV and MTT	Manual 2D	–	Eight parameters derived from the multiple MR sequences: CE-T1, T2, FLAIR, PD, ADC, rCBF, rCBV and MTT.	rCBV	OC-SVMs	Greater value of advanced MRI DWI and PWI derived measures as compared to conventional imaging for discrimination of RN from viable tumour
Tiwari et al.^26^	58 (training *n* = 43, test *n* = 15; GBM)	T1-CE, T2, FLAIR	Manual 2D	Matlab	119 texture maps: Haralick, Laws, Laplacian pyramid, histogram of gradient orientations	GLCM and Laws features in the lower Laplacian scale	SVM	Laplacian pyramid features were identified to be most discriminative, possibly because these emphasize edge-related differences between RT and RN at lower resolutions.
Skogen et al.^58^	95 (HGG = 68, LGG = 27)	3 T/T1-CE	Manual 2D	TexRAD^b^	Histogram metric	Fine textures scale	–	LGGs and HGGs were best discriminated using SD at fine-texture scale, with a sensitivity and specificity of 93% and 81% (AUC = 0.910, *P* < 0.0001)
Xie et al.^60^	42 (HGG = 27, LGG = 15)	3 T/dynamic contrast-enhanced	Manual 2D	OmniKinetics^c^	Five GLCM features - energy, entropy, inertia, correlation, and inverse difference moment (IDM)	Entropy and IDM	–	Evaluated five GLCM features from (DCE)-MRI of 42 patients with gliomas. They reported that entropy (AUC = 0.885) and IDM (AUC = 0.901) were able to differentiate grade III from grade IV and grade II from grade III gliomas, respectively.; no feature was able to distinguish subtypes of grade II and grade III gliomas.
Qi et al^[.126^	39 (HGG = 26, LGG = 13)	3 T/DWI/DKI	Manual 2D	ImageJ^d^	Histogram metric	Mean kurtosis (MK)	–	Histogram parameters on DKI were significant in differentiating high- (grade III and IV) from low-grade (II) gliomas (*P* < .05); mean kurtosis was the best independent predictor of differentiating glioma grades with AUC = 0.925
Tian et al.^59^	153 (Grade II = 42, III = 33, IV = 78)	3 T/Multiparametric (T1WI, T1-CE, T2WI, DWI, ASL)	Manual 2D-VOI	Matlab	GLCM, GLGCM histogram mean	30 and 28 Optimal features of 420 texture and 90 histogram features	SVM-RFE	Texture features were statistically significant over histogram parameters for glioma grading; AUC for classifying LGGs versus HGGs was 0.987, while it was 0.992 for grade III versus IV gliomas
Zacharaki et al.^96^	102 (mets = 24, meningiomas = 4, grade II gliomas = 22, grade III gliomas = 18, GBMs = 34)	3 T (T1, T2, FLAIR, DTI, perfusion)	Manual 2D	–	161: tumour shape, image intensity, Gabor texture	Different features for each pair-wise classification task, mainly comprising intensity from T1, T2, rCBV statistics and Gabor texture from FLAIR	LDA, kNN, SVM	The binary SVM classification achieved via a leave-one-out cross-validation reported accuracy, sensitivity, and specificity of 85%, 87%, and 97% for discrimination of metastases from gliomas and 88%, 85%, and 96% for discrimination of high-grade (grade III and IV) from low-grade (grade II) neoplasms.
Suh et al.^61^	77 (GBM = 23, PCNSLs = 54)	3 T/T1-CE, T2, FLAIR	Manual 2D	Python package, PyRadiomics 1.2.0^e^	Shape, volume, 1^st^ order, GLCM, GLRLM, mGLSZM, and wavelet transform	A total of 6366 radiomics features subjected to recursive feature elimination and random forest analysis with nested cross-validation	SVM	In comparing diagnostic performances, the AUC (0.921) and accuracy (90%) of the radiomics classifier was significantly higher than those of the 3 radiologists (*P* < 0.001)
Beig et al.^127^	medulloblastomas (*n* = 22), ependymomas (*n* = 12), and gliomas (*n* = 25)	T1, T2, FLAIR	Manual 2D	Matlab	52 CoLlAGe features	sum variance and entropy of CoLlAGe on T2	RF	Medulloblastomas exhibited higher CoLlAGe entropy values than ependymomas and Gliomas for the paediatric brain tumour cases.

*OC* one class, *SVM* support vector machine, *RFE* recursive feature elimination, *RF* Random Forest, *rCBV* relative cerebral blood volume, *RLM* run-length matrix, *ADC* apparent diffusion coefficient, *EC* edge contrast, – not available, *GLSZM* grey-level size-zone matrix, *GLCM* grey level co-occurrence matrix, *GLRLM* grey level run-length matrix features, *LDA* linear discriminant analysis, *S* sharpness, *K*_*T*_ measure of the total curvature, *VOI* volume of interest, *kNN* k-nearest neighbours.

^a^MathWorks, Natick, Massachusetts.

^b^https://imagingendpoints.com/texrad-software/.

^c^http://www.omnikinetics.com/.

^d^National Institutes of Health, Bethesda, Maryland.

^e^https://pyradiomics.readthedocs.io/en/latest/modules/radiomics/ngtdm.html.

**Table 2:** Prognostic applications.

Author	No. of patients	Magnet strength/MRI sequence	Segmentation manual vs automatic	Texture software	Type of radiomic analysis	Best discriminating features	Machine learning/statistical approach	Results
Beig et al.^87^	115 (GBM)	T1WI, T2WI, FLAIR	Manual 2D	Matlab	30 features: Laws, Gabor, GLCM	Laws energy (R5R5, E5E5, S5S5) from enhancing tumour and oedema	RF model	Features from the enhancing and oedematous regions were predictive of the extent of hypoxia. Features on the validation set were also found to be prognostic of overall survival
Kickingereder et al.^88^	119 (GBM)	3 T/T1-CE, FLAIR	Manual 3D	Medical Imaging Toolkit^a^	Histogram volume and shape features; texture features; wavelet analysis	11 features of 12,190 features	SPC analysis	SPC analysis performed better than clinical (age and Karnofsky Performance Score) and radiologic (rCBV and ADC) parameters
Molina et al.^128^	79 (GBM)	1.5–3 T/T1-CE	Manual 3D	Matlab	Five GLCM features, 11 GLRLM features	GLCM (entropy, homogeneity, contrast, dissimilarity) GLRLM (LRE, HGLRE, LRHGE, RPC)	Kaplan–Meier curves and Cox-proportional hazards analysis	Patients had better prognosis when high LRHGE, low RPC, low entropy, high homogeneity, and low dissimilarity were present (*P* < 0.05)
Chaddad et al.^129^	73 (GBM)/ TCIA	T1-CE, FLAIR	Semi-automatic 2D	–	GLCM JIM	JIM (entropy, inverse-variance) in necrosis region and (entropy, contrast) in oedema region	RF model	JIM of T1-CE and FLAIR images significantly predicted survival outcomes with moderate correlation; nine features were found to be associated with glioblastoma survival, *P* < 0.05, accuracy of 68%-70%; AUC of 77.56% with *P* = 0.003 was achieved when combining JIM, GLCM, and gene expression features into a single radiogenomic signature.
Liu et al.^130^	119 (GBM)/TCGA	T1WI, T2WI, FLAIR, T1-CE	Manual 2D	–	Histogram, GLCM, GLRLM	13 textural features	SVM-RFE	T1-CE sequence performed best, with AUC of 0.7915 and accuracy of 80.67%
Yang et al.^131^	82 (GBM)/TCGA	T1-CE, T2WI, FLAIR	Manual 3D	Matlab	SFTA, RLM, LBP, HOG, Haralick texture features	–	RF model	Molecular subtypes and 12-month survival was predicted by several features; SFTA features on T1-CE were most predictive of survival and proneural subtype (AUC = 0.82); RLM features on T2 FLAIR axial for neural (AUC = 0.75); Haralick features on T1-CE for classic (AUC = 0.72); HOG features on T2 FLAIR axial for mesenchymal (AUC = 0.70)
Bahrami et al.^95^	33 (HGG)	3 T/FLAIR	Semi-automatic 3D	–	EC	–	Kaplan–Meier curves	Reported that lower EC of the FLAIR hyperintense region was associated with poor PFS (*P* = 0.009) and OS (*P* = 0.022) status post-bevacizumab therapy
Jain et al.^89^	45 (GBM)	1.5/3 T, T1, T2w/perfusion, FLAIR	Manual 2D	R^b^	VASARI features, rCBV statistics	rCBV_NER_	Random Survival Forest	Increased maximum rCBV_CER_ found to be associated with increased risk of death
McGarry et al.^90^	81 (GBM)	1.5/3 T, T1, CE-T1, T2, FLAIR	Semi-automatic 3D	–	81 radiomic profiles (5 RPs): 4-digit code assigned to each voxel representing the intensity-based segmentation	Five RPs correlated with survival when thresholded by volume	Cox Regression	Presented a method for creating radiographic profiles by combining intensity information from multiple MRI scans. Pathologically validated that voxels indicated by one of the RPs contained hypercellular tumour and necrosis.
Lao et al.^117^	112 (GBM)	1.5/3 T, T1, T1-CE, T2, FLAIR	Manual 2D	Python CAFFE	1403 handcrafted (HC) features: geometry, intensity, texture and 98304 deep features using transfer learning	150 HC and deep features	LASSO Cox Regression	The radiomics signature achieved a C-Index of 0.731 for the discovery dataset, and 0.710 for the independent validation set.
Li et al.^132^	92 (GBM)/TCIA, local data	T1, T1-CE, T2, FLAIR	Automated using Matlab	R^b^	Texture at different voxel size, quantization and grey levels	-	Cox regression model	The multiparametric signature achieved better performance for OS prediction (C-Index = 0.705)
Sun et al.^133^	542 (training *n* = 285; validation =66; test=191; LGG,HGG)/BRATS 2018	T1, T1-CE, T2, FLAIR	3D CNN architectures	Pyradiomics toolbox	Shape, texture, first-order statistics	Age, 14 selected features	RF regression	Achieved 61% accuracy in predicting survival outcome.
Sanghani et al.^134^	163 (GBM)/BRATS 2017	T1, T1-CE, T2, FLAIR	Segmentation masks from BraTS 2017 dataset	–	Texture, shape, volumetric, age	Top 150 selected features out of 2200	SVM-RFE/fivefold cross-validation	The 2-class and 3-class OS group prediction accuracy obtained were 98.7% and 88.95% respectively.
Beig et al.^135^	460 (GBM), Male = 290, female = 170	T1, T1-CE, T2, FLAIR	Semi-automatically with Slicer 3D^d^	Matlab	2850 features: Laws, Gabor, shape based	Five Laws energy, two Gabor wavelets; one shape based	Cox regression model	Sexually dimorphic radiomic risk score (RRS) models that are prognostic of overall survival (OS) in primary GBM
Han et al.^136^	178 (HGG)/TCIA, local dataset	T1-CE	Manual segmentation	Matlab, Keras^c^, R	348 handcrafted - volume, size, texture, intensity, first-order statistical + 8192 deep CNN features	–	Elastic net-Cox modelling	The combined feature analysis framework classified the patients into long- and short-term survivor groups with a log-rank test *P* < 0.001

*EC* Edge contrast, *JIM* joint intensity matrices, *RF* random forest, *TCGA* The Cancer Genome Atlas, *TCIA* The Cancer Imaging Archive, *LRE* long-run emphasis, *HGLRE* high grey-level run emphasis, *LRHGE* long-run high grey-level emphasis, *RPC* run percentage, *SFTA* segmentation-based fractal texture analysis, *LBP* local binary pattern, *HOG* histogram-oriented gradient, *SPC* supervised principal component.

^a^http://www.mitk.org/wiki/The_Medical_Imaging_Interaction_Toolkit_(MITK).

^b^R statistical and computing software (http://www.r-project.org).

^c^Keras (www.keras.io).

^d^http://www.slicer.org.

**Table 3:** Applications in selecting optimal therapy.

Author	No. of patients	Magnet strength/MRI sequence	Segmentation manual vs automatic	Texture software	Type of radiomic analysis	Best discriminating features	Machine learning/statistical approach	Results
Beig et al.^137^	83 LGGs	T2w/FLAIR	Manual 2D	Matlab	GLCM, Gabor	3D Gabor	SVM	Initial results indicate that radiomic features from non-enhancing regions on T2 and infiltrative edges on FLAIR can segregate the 3 subgroups.
Zhang et al.^63^	152 (IDH mutant = 92, wild-type = 60)	1.5T–3 T/T1-CE, T2WI, FLAIR	Manual 3D	Matlab	GLCM GLGCM	15 Optimal features from 168 Haralick features	SVM-RFE	AUC 0.841 and accuracy of 82.2% for non-invasively discriminating *IDH* mutation of patient with glioma
Hsieh et al.^138^	39 (IDH mutant = 7, wild-type = 32; TCGA)	1.5T–3 T/T1-CE	Manual 2D	CAD system	Morphological intensity-GLCM	14 GLCM	Binary logistic regression classifier	Textural features describing local patterns yielded an accuracy of 85% in detecting the IDH status
Han et al.^64^	42 (IDH mutant = 21, wild-type = 21)	3 T/T1WI, T2WI, 3D-T1-CE	Manual 3D	OmniKinetics	29 Texture features from first-order and GLCM	Inertia, cluster prominence, GLCM entropy	–	Showed joint variables derived from T1WI, T2WI, and contrast-enhanced T1WI imaging histograms and GLCM features could be used to detect IDH1-mutated gliomas. The AUC of Joint Variable_T1WI+C_ for predicting IDH1 mutation was 0.984, and the AUC of Joint Variable_T1WI_ for predicting the IDH1 mutation was 0.927
Jakola et al.^65^	25 (IDH mutant = 20, wild-type = 5)	3 T/3D-FLAIR	Semiautomatic 3D	ImageJ	Homogeneity, energy, entropy, correlation, inertia	Homogeneity	–	Homogeneity discriminated patients with LGG in *IDH* mutant and *IDH* wild-type (*P* = 0.005), AUC for combined parameters was 0.940 for predicting IDH mutation; authors could not separate *IDH*-mutant tumours on basis of 1p/19q-codeletion status
Bahrami et al.^66^	61 (IDH mutant = 43, wild-type = 11); 7 unknown)	3 T/Pre- and post-T1-CE, FLAIR	Semiautomatic 3D	3D-co-occurrence matrix	Histogram, GLCM	Homogeneity, pixel correlation, EC	Logistic regression with LASSO regularization	Greater signal heterogeneity and lower EC noted in *IDH* wild-type tumours; *IDH* mutant tumours with 1p/19q-codeleted status; lower EC in MGMT-methylated tumours
Shofty et al.^139^	47 LGGs	1.5T–3 T/FLAIR, T2, TI-CE	Automatic using FSL^a^, 3D	Matlab	Histogram, contrast, correlation, energy, entropy, homogeneity	39 of 152 textural features	17 classifiers	Ensemble of bagged trees classifier achieved the best performance (Accuracy = 87%; AUC = 0.87) for the detection of 1p/19q codeletion; majority of differences detected for T2 and T1-CE
Kickingereder et al.^94^	172 (GBM)	3 T/Pre- and post-T1-CE, FLAIR	Semiautomatic 3D	Medical imaging Toolkit^b^	188 imaging features, 17 first-order features (FO), 9 volume and shape features (VSF) and 162 texture features (GLCM,) GLRLM).	–	Supervised principal component (superpc) analysis	The superpc predictor stratified patients in the validated set into a low or high-risk group for PFS (HR = 1.85, *P* = 0.030) and OS (HR = 2.60, *P* = 0.001).
Grossmann et al.^100^	126 (GBM)	Baseline and follow-up MRI (1 and 6 wks), T1WI, T2WI, FLAIR, T1-CE	Semi-automatically with Slicer 3D^d^	R version 3.1.0^c^	First-order statistics of the voxel intensity histogram; tumour shape; tumour texture	Information correlation	PCA	Radiomics provides prognostic value for survival and progression in patients with recurrent glioblastoma receiving bevacizumab treatment; features derived from postcontrast T1WI yielded higher prognostic power compared with T2WI
Wu et al.^140^	126 (grade II-III = 43 and grade IV = 83)/TCIA	T1, T1-CE, T2, FLAIR	Semiautomatic approach	R version 3.3.1^c^	GLCM texture, Volume, intensity, histogram, diffusion	20 of 704 radiomic features	SVM, kNN, RF, NB, NN, FDA, Adaboost/tenfold cross-validation	Random Forest (RF) showed high predictive performance for identifying IDH genotype (AUC = 0.931, accuracy=88.5%)
Zhou et al.^141^	744 (LGG, HGG)/ TCIA, local datasets	T1-CE, T2-FLAIR	Semi-automatically with Slicer 3D^d^	Matlab	Histogram, texture, age, shape	Top 15 features out of 127	RF/Train-test model	The overall accuracy for 3 group prediction (IDH-wild type, IDH-mutant and 1p19q co-deletion, IDH-mutant and 1p19q non-codeletion) was 78.2%
Lee et al.^142^	123 (GBM)	T1, T2, T1-CE, FLAIR, PWI, DWI	Manual segmentation	Nordi-cICE	Volume, ADC map, CBV	Four of 31 radiological features	kNN, SVM, RF, Adaboost, decision tree, NB, LDA, gradient boosting	Prediction rate of IDH1 mutation status with 66.3–83.4% accuracy
Sudre et al.^143^	333 (IDH mutant = 151, wild-type = 182);	1.5 T/T1, T2, FLAIR, DSC MRI	Olea Sphere, Version 3	–	Shape, intensity, texture	Nine histogram features, 11 texture features	RF/cross-validation	Gliomas were correctly stratified 53% for grade classification and 71% for IDH classification

*GLCM* grey level co-occurrence matrix, *GLRLM* grey level run-length matrix features, *LDA* linear discriminant analysis, *PCA* principal component analysis, *Adaboost* adaptive boosting, *NB* Naive Bayes, *FDA* flexible discriminant analysis, *NN* neural network.

^a^FSL (http://www.fmrib.ox.ac.uk/fsl).

^b^http://www.mitk.org/wiki/The_Medical_Imaging_Interaction_Toolkit_(MITK).

^c^R statistical and computing software (http://www.r-project.org).

^d^http://www.slicer.org.

## Discussion

Conventional structural MRI, although popular as a universally available imaging modality, has often failed in distinguishing tumour recurrence, pseudoprogression and radionecrosis with classic criteria such as enhancement, mass effect and perilesional oedema.^11^ The enhancement patterns like ‘soap bubble’, ‘Swiss cheese, and moving wave front are considered therapy-induced, but they lack reproducibility and mandate further texture analysis, spectroscopy or blood flow studies for validation.^96^ The dilemmas and delays in detection of progression versus pseudoprogression versus mixed lesions can delay treatment or cause discontinuation of treatment.^26,36^

Presently, stereotactic brain biopsy remains the gold standard for histological and genetic classification. However, the high tumour heterogeneity in gliomas may decrease the accuracy of biopsies and render pathological diagnosis inconclusive in about 7–15% of patients.^97^ The ability to assess local imaging presentations of tumours, based on the underlying genotype, could potentially mitigate bias associated with tissue sampling during biopsy procedures. The potential for radiomic analysis to distinguish glioma molecular subtypes non-invasively would not only provide additional prognostic information, but would also assist in the selection of targeted chemotherapy in patients with multiple genetic mutations and potentially high-grade tumour types.^84^

The median survival of glioblastoma with surgical debulking is 15 months, with the clinical outcome depending on the extent of initial resection and response to chemo-radiation therapy.^98^ Radiomic risk models can therefore be utilised to better predict treatment response, PFS and OS. By obtaining the radiogenomic profile of a tumour non-invasively,^99^ the effect of anti-angiogenic therapies, such as bevacizumab, can be assessed without harm to the patient.^100^ To date, the efficacy of anti-angiogenic therapies is primarily monitored with MRI and MRS. Tumour pseudo response to anti-angiogenic therapies becomes problematic in that standard enhancement characteristics of a tumour may appear falsely reduced after the administration of anti-angiogenic medications.^101^ The rapid decrease in contrast enhancement and vasogenic oedema suggests anti-angiogenic response when the tumour may be stable or has progressed. The alterations in MR characterisation of tumours after anti-angiogenic therapy is primarily related to changes in blood–brain barrier permeability. By utilising radiomics in such cases, the actual tumour response may be monitored, and treatment strategies can be further tailored prior to tumour progression.

Gliomas are genetically highly heterogenous. The broad genetic alterations, coupled with microenvironment biochemistry, manifest in characteristic appearances both on gross histology and on the radiological scale. In addition to the work reviewed in the boarder context of IDH, MGMT and EGFR differences, other associations have also been supported. Contrast enhancement has been shown to be associated with genes implicated in hypoxia–angiogenesis pathway,^102^ such as vascular endothelial growth factor (VEGF). Radiomic attributes capturing abnormal intensity patterns in the internal capsule have been shown to be correlated with MYC oncogene expression.^103^ Interestingly, multiple radiogenomic correlation experiments have revealed strong associations of imaging phenotypes with pathways that are implicated in extracellular matrix destruction, cell invasion and metabolism.

Furthermore, radiomics offers an opportunity to perform an analysis on complete tumour that could mitigate the limitation of sampling errors and inability of complete molecular and histopathological assessment by neuropathologists given the lack of tumour sample.^104–106^ With quantitative mutation values rather than binary designations, radiomics can help neuro-oncologists and neurosurgeons make personalised therapy decisions and reliably predict response to therapies.

### Limitations

A major feature limiting radiomic quantification is poor reproducibility secondary to variability and lack of consistency attributed to the absence of standardised acquisition parameters and radiomic approaches.^107^ The accuracy of radiomic signatures typically varies when tested on different datasets. Multiple studies have addressed impact of different acquisition parameters on textural analysis. Magnet strength, flip angles, different spatial/matrix size, TR/TE variations in T1WI and T2WI, and different scanner platforms can affect texture features.^108–112^ Molina et al.^112^ found that no textural measures were robust under dynamic range changes, entropy was the only robust feature under spatial resolution changes. Buch et al.^109^ concluded that some of the features were more robust and some of the features were more susceptible to different acquisition parameters, necessitating the need for standardised MRI techniques for textural analysis. Furthermore, variation in usability of textural analysis software add complexity to standardisation and reproducibility. Multiple studies used indigenous software with varying algorithms making reproducibility and repeatability of these studies almost impossible. Future studies are needed to assess accuracy of these results from different type of software to help with standardisation.

Scarcity of publicly available databases with annotated radiological studies for specific clinical domains limit capability of researchers to conduct large sample size studies. Small sample size and a high number of prediction variables often leads to overfitting, a major limitation in machine learning models. To prevent overfitting, it is recommended to have sample size 6–10 times larger than the analysed variables or conducting analysis with a few preselected robust variables only. Collaboration among research universities is required to create professionally annotated standardised datasets for larger cohort studies which can be split into training, testing, and validation datasets to avoid overfitting. This would also allow the researchers to test their algorithms on external cohorts and validating robustness of their solutions. A recent development towards achieving this is the use of federated learning which facilitates multi-institutional validation of machine learning models without explicit sharing of data using a distributed framework.^113^

Variability in the selection of appropriate regions of interest for feature extraction can affect certain radiomic attributes, such as shape-based measures. There are no existing guidelines for radiologists to report quantitative imaging features, making huge existing image repositories inaccessible for curation. For generating high-quality data with segmented and annotated appropriate regions of interest, radiologists need to be integral part of data quantification and curation.^114^

The lack of routinely acquired gene expression profiles and tissue sampling errors impose limitations to the application of radiogenomics in current clinical workflow.^115^ It is difficult for a single institute to create a large imaging database with auxiliary data such as genomic profile, demographics, treatment information and their outcomes. The Cancer Genome Atlas (TCGA) has made cancer datasets publicly available with a comprehensive catalogue of genomic profiles to address this issue. The clinical translation of radiogenomics is also hindered by spatial and temporal heterogeneity within a given brain tumour. However, ability of radiomics to perform an analysis on complete tumour might address this limitation.

Deep learning algorithms that facilitate automated feature learning have recently shown great promise in tasks ranging from tumour segmentation^116^ to survival prediction.^117^ Methods combining radiology and pathology datasets have been proposed to distinguish gliomas into oligodendroglioma and astrocytoma classes.^118^ Such methods, however, require large datasets for training purposes besides often lacking transparency and interpretability. Uncertainty and interpretability of deep learning networks in the field of medical imaging is an active area of research. A significant challenge for the translation of radiomics and deep learning algorithms into clinical workflow as clinical decision support systems stems from a regulatory perspective. US Food and Drug Administration (FDA) has closely regulated CAD (computer-aided detection) systems that rely on machine learning and pattern-recognition techniques; machine learning models present new regulatory challenges and require specialised guidance for submissions seeking approval (https://www.fda.gov/medical-devices/software-medical-device-samd/artificial-intelligence-and-machine-learning-software-medical-device). Furthermore, new AI models keep evolving even after going to market as they are exposed to more data. It is vital to adopt periodic testing requirements over specific time intervals to make sure the adaptive changes of these models follow forecasted projections.

### Future directions

Recent initiatives such as the image biomarker standardisation initiative have proposed certain guidelines based on results obtained on radiomics phantoms.^119^ Test–retest experimental settings have also been widely proposed to facilitate selection of stable and robust radiomic measures. In one of the first studies of this kind, the repeatability of CT radiomics was ascertained in a “coffee-break” test–retest setting with scans obtained from the same scanner within an interval of 15 min.^120^ Similar settings are warranted for brain imaging to identify suitable radiomic features for clinical applications. The Cancer Imaging Archive hosts imaging datasets of brain tumour collections (HGGs and LGGs), among other cancers, obtained from several institutions. Such datasets have been widely used by the research community to develop and validate radiomics and radiogenomics tools.

One obvious deficiency in virtually all the retrospective radiogenomic studies is lack of information regarding the location of the biopsy sample vis-à-vis pixels in the patient’s images.^121^ Image-localised biopsies and subsequent imaging-pathology co-registration are essential steps in mitigating biases associated with locating biopsy region on MRI. Hu et al.^122^ have previously co-registered MRI scans and corresponding texture maps with biopsy locations to study regional genetic variation with spatially matched imaging descriptors. In a follow-up study, Hu et al.^123^ have proposed a Gaussian process and transductive learning based probabilistic model to quantify spatial uncertainty in radiogenomic pipelines. The sparse availability of ground truth labels in radiogenomic models can be modelled as a ‘weak supervision’ or ‘incomplete supervision’ task. Multi-instance learning techniques may be implemented to address this limitation.^124^

The field of radiomics promises to elevate the role of medical imaging by enabling objective tumour characterisation. In oncology, radiomics can provide prognostic information non-invasively via biomarker utilisation.^19^ In neurosurgery, it can be used for improved pre- and post-operative treatment planning. Emerging companies are now providing software that delivers web-based radiological analysis with the PACS viewing system.^125^ Support from academic institutions, such as the American College of Radiology, is growing. Such efforts can readily facilitate the transition of radiomic research to clinical practice.

## Conclusions

Radiomics is not intended to replace radiologists in the future, but rather improve disease diagnosis and characterisation with greater precision. It is imperative for the future of neuroradiology, neurosurgery and neuro-oncology to utilise advances in radiomics and radiogenomics in order to provide less invasive and tumour-specific precision treatment strategies and to ultimately optimise patient care. In order for this field to continue evolving and make its way into clinical practice, it is vital to develop more standardised and reproducible methods of data interpretation, maintain publicly available databases of radiological studies, and conduct prospective large-scale multi-institutional clinical trials. In the future, the fields of radiomics and radiogenomics promise to improve the utility of already available imaging modalities and channel them towards personalised medicine.

## Data Availability

Not applicable.
